# The rise of diversity in metabolic platforms across the Candidate Phyla Radiation

**DOI:** 10.1186/s12915-020-00804-5

**Published:** 2020-06-19

**Authors:** Alexander L. Jaffe, Cindy J. Castelle, Paula B. Matheus Carnevali, Simonetta Gribaldo, Jillian F. Banfield

**Affiliations:** 1 Department of Plant and Microbial Biology, University of California, Berkeley, Berkeley, CA USA; 2 Department of Earth and Planetary Science, University of California, Berkeley, Berkeley, CA USA; 3 Chan Zuckerberg Biohub, San Francisco, CA USA; 4 Department of Microbiology, Unit Evolutionary Biology of the Microbial Cell, Institut Pasteur, Paris, France; 5 Department of Environmental Science, Policy, and Management, University of California, Berkeley, Berkeley, CA USA; 6 Innovative Genomics Institute, University of California, Berkeley, Berkeley, CA USA

**Keywords:** Candidate Phyla Radiation, Metabolic evolution, Lateral gene transfer, Bacterial carbon metabolism, NiFe hydrogenase, Phylogenomics, Comparative genomics

## Abstract

**Background:**

A unifying feature of the bacterial Candidate Phyla Radiation (CPR) is a limited and highly variable repertoire of biosynthetic capabilities. However, the distribution of metabolic traits across the CPR and the evolutionary processes underlying them are incompletely resolved.

**Results:**

Here, we selected ~ 1000 genomes of CPR bacteria from diverse environments to construct a robust internal phylogeny that was consistent across two unlinked marker sets. Mapping of glycolysis, the pentose phosphate pathway, and pyruvate metabolism onto the tree showed that some components of these pathways are sparsely distributed and that similarity between metabolic platforms is only partially predicted by phylogenetic relationships. To evaluate the extent to which gene loss and lateral gene transfer have shaped trait distribution, we analyzed the patchiness of gene presence in a phylogenetic context, examined the phylogenetic depth of clades with shared traits, and compared the reference tree topology with those of specific metabolic proteins. While the central glycolytic pathway in CPR is widely conserved and has likely been shaped primarily by vertical transmission, there is evidence for both gene loss and transfer especially in steps that convert glucose into fructose 1,6-bisphosphate and glycerate 3P into pyruvate. Additionally, the distribution of Group 3 and Group 4-related NiFe hydrogenases is patchy and suggests multiple events of ancient gene transfer.

**Conclusions:**

We infer that patterns of gene gain and loss in CPR, including acquisition of accessory traits in independent transfer events, could have been driven by shifts in host-derived resources and led to sparse but varied genetic inventories.

## Background

Metagenomics approaches have been extremely fruitful in the discovery of new lineages across the tree of life [1–4]. Genomes recovered from poorly represented or novel groups have helped greatly to elucidate the evolutionary processes contributing both to broad bacterial and archaeal diversity and also to the distribution of metabolic capacities over various lineages [5–7].

The Candidate Phyla Radiation is a large group of bacterial lineages that lack pure isolate cultures and have been primarily defined through genome-resolved metagenomics [1, 8]. While estimates vary depending on the methods used [9, 10], CPR bacteria are predicted to constitute a significant portion of bacterial diversity that is distinct and divergent from other groups [11]. Additionally, CPR bacteria generally have relatively small genome and cell sizes, have extremely reduced genomic repertoires, and often lack the capacity to synthesize lipids, amino acids, and nucleotides [1, 8, 12]. The CPR may have diverged early from other bacteria and subsequently diversified over long periods of time, or they may have arisen via rapid evolution involving genome streamlining/reduction [13]. Arguing against recent diversification from other bacteria are the observations that CPR bacteria do not share genomic features associated with recent genome reduction, have uniformly small genomes, cluster independently from other metabolically reduced symbionts, and possess metabolic platforms consistent with projections for the anaerobic environment of the early Earth [13–15].

Recently, an analysis of entire proteomes showed that genetic capacities encoded by CPR bacteria are combined in an enormous number of different ways, yet those combinations tend to recapitulate inferred phylogenetic relationships between groups [15]. These analyses also revealed that some lineages have relatively minimal core gene sets compared to others within the CPR [5, 15], suggesting variation in the degree of genome reduction across the radiation. Additionally, previous work has shown that lateral gene transfer probably underlies distributions of specific protein families in CPR bacteria, including RuBisCO [16, 17]. The observation that organisms from this group also encode genes for nitrogen, hydrogen, and sulfur compound transformations at a low frequency [5, 18–21] raises the possibility that these capacities may also have been shaped by lateral transfer. Overall, the extent to which lateral transfer, genomic loss, and vertical transfer have interacted to shape evolution of metabolic repertoires across the CPR is still unknown [13].

Here, we integrate insights from CPR bacterial genomes from diverse environments with a robustly resolved internal phylogeny to investigate the processes governing the evolution of metabolic pathways in this group. A key aspect of our approach was the development of custom cutoffs for HMM-based metabolic annotation that are sensitive to the divergent nature of proteins from CPR organisms. We investigated central carbon metabolism (glycolysis and the pentose phosphate pathway), hypothesizing that these pathways may be primarily shaped by vertical inheritance, as well as sparsely distributed traits (nitrogen, hydrogen, sulfur metabolism) that we predicted were shaped by lateral transfer. Mapping of metabolic capacities onto the reconstructed reference tree and gene-species tree reconciliations showed that a mixture of vertical inheritance, gene loss, and lateral transfer have differentially shaped the distribution of functionally linked gene sets. Information about the evolution of gene content may help to shed light on evolutionary scenarios that shaped the characteristics of extant CPR bacteria.

## Results

### A robust reference phylogeny for the CPR

**Fig. 1:**
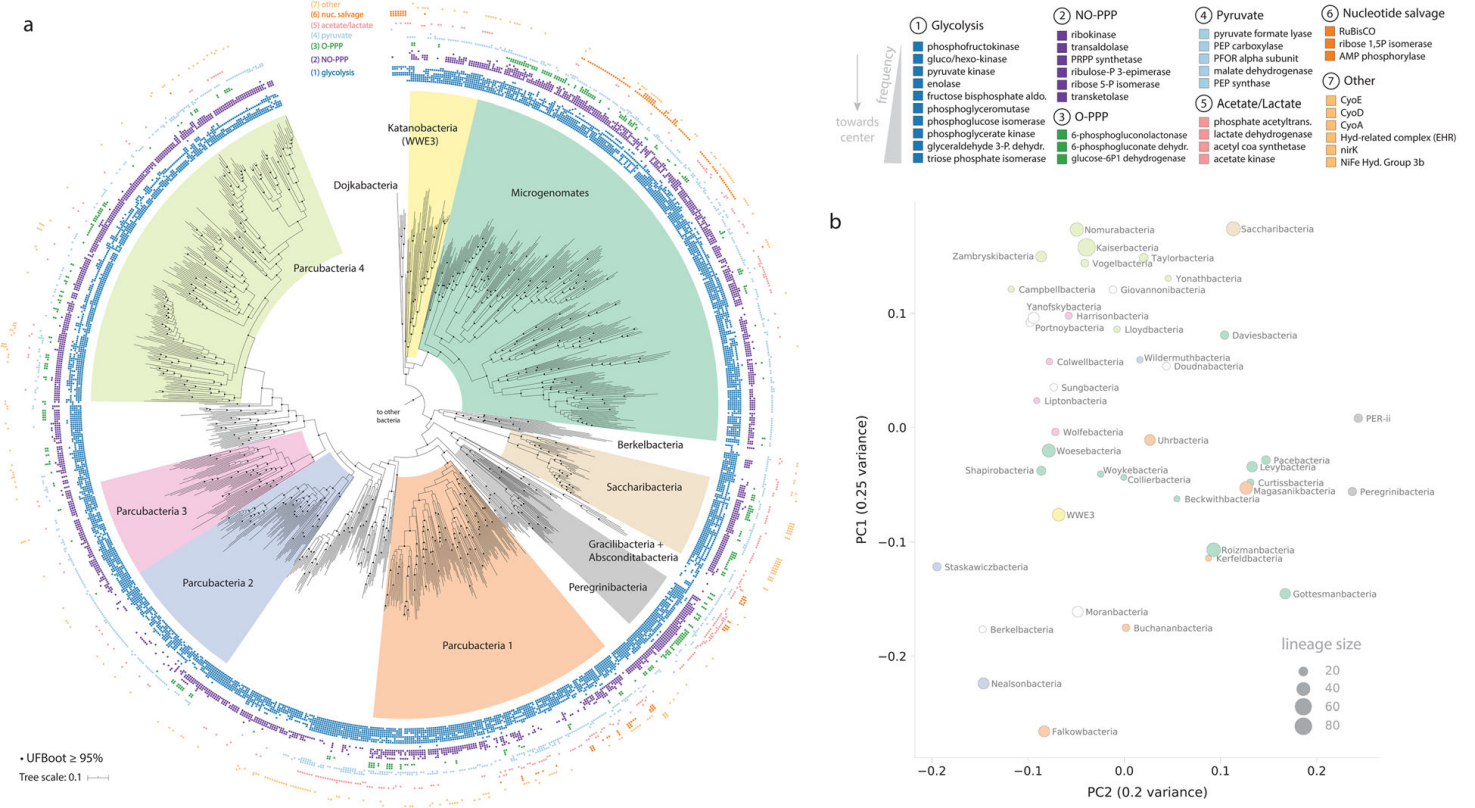
Phylogenetic relationships and metabolic similarity among Candidate Phyla Radiation bacteria. **a** Maximum-likelihood tree based on the concatenated set of 16 ribosomal proteins (1427 amino acids, LG+R10 model). Scale bar represents the average number of substitutions per site. Monophyletic subgroups within the Parcubacteria also supported in the concatenated RNA polymerase tree are indicated as Parcubacteria 1–4. The presence/absence of a subset of targeted metabolic traits is indicated as concentric rings. Abbreviations: aldo., aldolase; dehydr., dehydrogenase, PRPP, phosphoribosylpyrophosphate, PEP, phosphoenolpyruvate; PFOR, pyruvate:ferredoxin oxidoreductase; acetyltrans., acetyltransferase; Hyd, hydrogenase. Fully annotated trees with all included lineages are available in Additional file 2, Fig. S2. **b** Principal coordinates analysis describing similarity between metabolic platforms of CPR lineages with 8 or more representative genomes

We gathered a large set of curated genomes of CPR bacteria from diverse environments, including both previously published and newly assembled sequences (the “Materials and methods” section). Quality filtration of this curated genome set at ≥ 70% completeness and ≤ 10% contamination and subsequent de-replication yielded a non-redundant set of 991 genomes for downstream phylogenetic and metabolic analysis (Additional file 1, Table S1). To improve recovery of phylogenetic markers from the collected set of genomes, we combined visualization of HMM bitscores with a phylogenetic approach to set sensitive, custom thresholds for two independent sets of markers composed of 16 syntenic ribosomal proteins (rp16) and the two RNA polymerase subunits (RNAp) (the “Materials and methods” section; Additional file 2, Fig. S1). Phylogenies based on these two marker sets were generally congruent for deep relationships within the CPR, with both trees supporting the distinction of CPR from the bacterial outgroup and the monophyly of the Microgenomates and Parcubacteria superphyla, respectively (Fig. 1a; Additional file 2, Fig. S2). Some clades were also supported by the absence of particular ribosomal proteins—the Microgenomates, along with the Dojkabacteria and Katanobacteria, lacked ribosomal protein L9 (rpL9), while a subset of Parcubacteria lacked the ribosomal protein L1 (rpL1), as observed previously [1]. Our results also suggested the presence of four generally well-supported (≥ 95% ultrafast bootstrap in three of four cases), monophyletic subgroups within the Parcubacteria (Fig. 1a, Parcubacteria 1–4). Although internal relationships between these subgroups varied slightly between trees (Additional file 2, Fig. S2), in both cases, Parcubacteria 1 (comprising 9 lineages) was the deepest clade, whereas Parcubacteria 4 (10 lineages) was the most shallow (Fig. 1a). Ten other lineages of Parcubacteria formed paraphyletic clades outside of these subgroups. We also show that Dojkabacteria (WS6), Katanobacteria (WWE3), Peregrinibacteria, Kazanbacteria, and Berkelbacteria are among the most deeply rooting clades outside the established superphyla (Fig. 1a).

### CPR bacteria encode variable and overlapping metabolic repertoires

We next leveraged the robust reference tree of the CPR to evaluate the distribution and combinations of capacities across the radiation. While CPR bacteria lack some core biosynthetic capacities, they do in fact possess numerous metabolic capacities involved in carbon, hydrogen, and possibly sulfur and nitrogen cycling [5, 12, 18, 20]. We focused on these traits for our subsequent analysis, reasoning that they are most likely to impact the ability of CPR bacteria to derive energy from organic compounds and contribute to biogeochemical transformations in conjunction with their hosts and other community members. To overcome the challenges inherent to metabolic annotation of divergent lineages and minimize the chance of false negatives, we extended our custom HMM thresholding approach to the selected set of biogeochemically relevant traits (the “Materials and methods” section; Additional file 2, Fig. S3; Additional file 3, Table S2) and mapped the resulting binary presence/absence profiles for specific functionalities onto the reconstructed rp16 tree. Looking across the selected traits, we observed a high degree of variation in the overall repertoires of lineages within the CPR, including some with extremely minimal metabolic complements like Dojkabacteria and Gracilibacteria. This is consistent with both observations from genomic studies of these lineages [12] as well as more recent insights examining entire proteomes [15].

An important open question is whether clades of CPR bacteria within broad phylogenetic groupings possess similar combinations of metabolic capacities. To investigate this, we used the distributions of the targeted traits to compute the frequency at which each trait was found within lineages. We then generated a distance matrix from the results and performed a principal coordinate analysis to visualize clustering of lineages based on the similarity of their overall metabolic platforms (Fig. 1b, the “Materials and methods” section). We reasoned that genes missing due to genome incompleteness could impact clustering, particularly for small lineages with only several members. Thus, we restricted the analysis to those groups with at least 8 member genomes. The results suggest that member lineages within some broad phylogenetic groupings are metabolically similar (e.g., Parcubacteria 3 and 4) but others clustered more closely with lineages that are distantly related. For example, lineages within the Microgenomates and Parcubacteria 1 were highly dispersed across the axes of variation (Fig. 1b), suggesting that member groups encode highly variable combinations of traits.

### Functionally linked metabolic genes display different evolutionary profiles

**Fig. 2:**
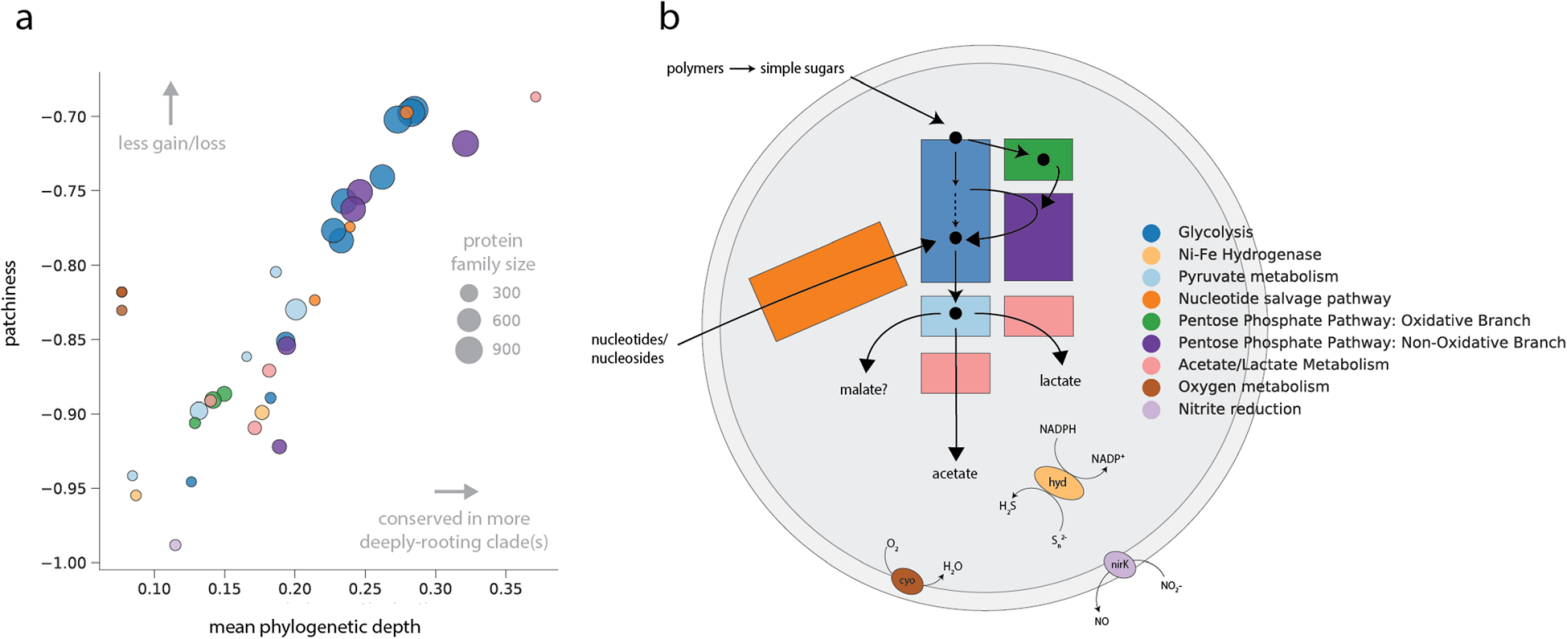
Metabolic traits encoded by CPR bacteria exhibit varying evolutionary profiles, including those in the same pathway (e.g., glycolysis genes). **a** Evolutionary profiles generated from phylogenetic depth and patchiness of gene distributions over the rp16 topology. Each point represents a metabolic gene shaded to match the functional category/pathway in **b**, schematic representing a generalized metabolic platform for CPR bacteria

The observation that distributions of traits are variable and potentially decoupled from phylogenetic relatedness raises the possibility that more complex, enzyme-specific patterns might underlie metabolic diversity in the CPR. To address this, we drew upon trait distributions to compute two metrics across the reference tree—the first to quantify the average branch length of clades in which a trait is conserved (phylogenetic depth) and the second to analyze trait patchiness, related to the number of gains/losses of a binary trait over a tree (the “Materials and methods” section) [22]. Generally, traits with a high phylogenetic depth correspond to those that are conserved in more deeply rooting clades, whereas traits with lower depth correspond to those that occur primarily among shallow clades. Similarly, high patchiness is expected when a given trait is more randomly dispersed across a clade, whereas traits with low patchiness scores correspond to those that are highly conserved within groups. These two metrics are therefore complementary and were integrated to create an “evolutionary profile” for each trait. Among CPR bacteria, we observed that an increase in phylogenetic depth generally correlates with a decrease in patchiness (Fig. 2a). High-depth traits also corresponded to larger protein families more frequently observed across the radiation (family size), though several smaller protein families (phosphate acetyltransferase, AMP phosphorylase, RuBisCO) reached relatively high phylogenetic depths because they were conserved in deeply rooting clades like the Dojkabacteria and Peregrinibacteria. On the other hand, hydrogen/sulfur metabolism, acetate/lactate metabolism, and the oxidative pentose phosphate pathway exhibited relatively high patchiness and low phylogenetic depth, consistent with their sparse but also wide distributions across distantly related groups (Fig. 2). Intriguingly, some traits displayed a relatively low phylogenetic depth but were less patchily distributed than would be predicted from the overall trend (e.g., genes involved in aerobic metabolism).

**Fig. 3:**
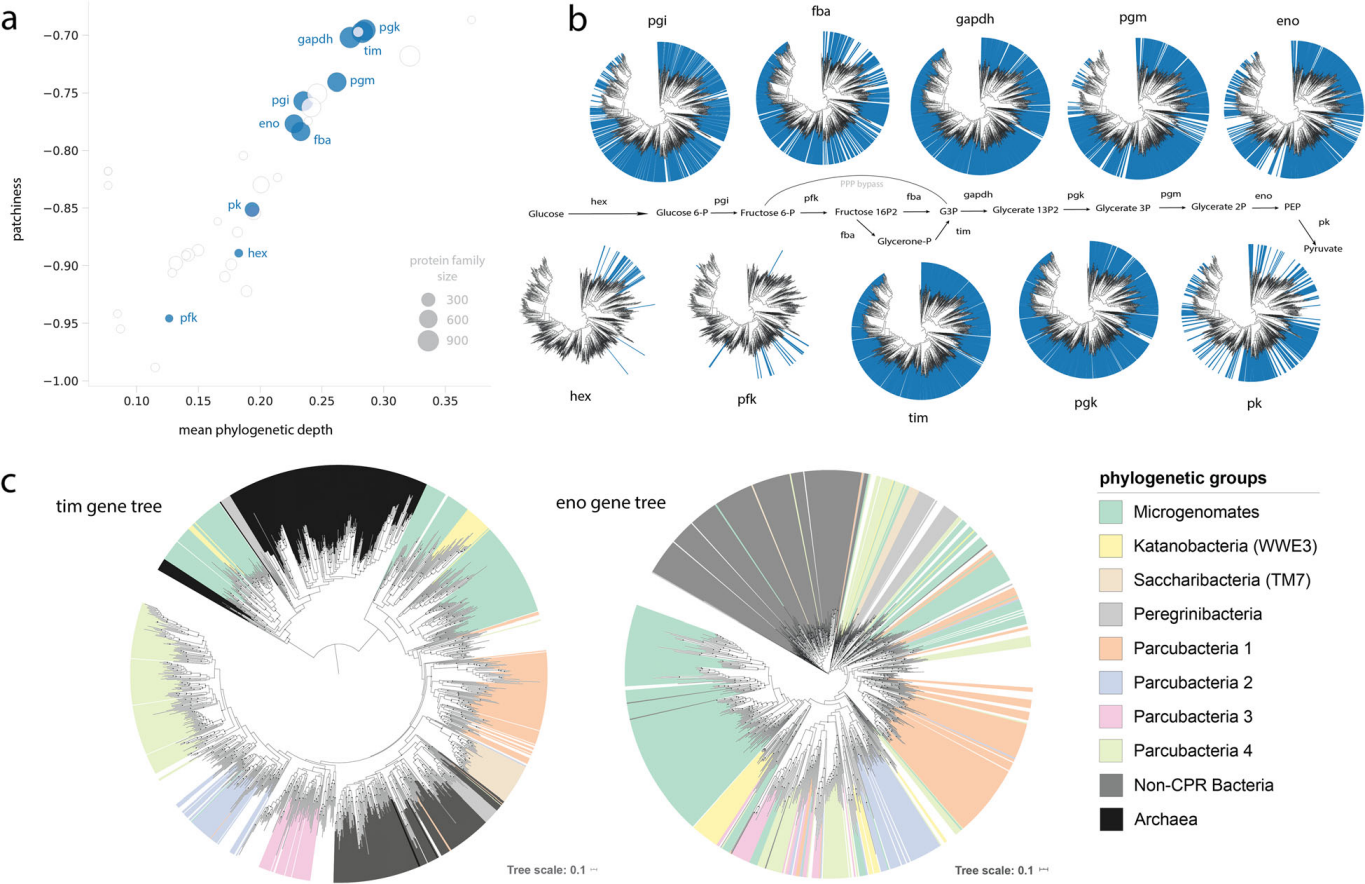
Patterns of distribution and gene trees for glycolytic enzymes across the CPR. **a** Evolutionary profiles based on patchiness and phylogenetic depth and **b** presence/absence profiles over the rp16 tree. **c** Protein-specific molecular phylogenies for triose phosphate isomerase (tim) and enolase (eno). Abbreviations: hex, hexokinase; pfk, phosphofructokinase; pk, pyruvate kinase; fba, fructose bisphosphate aldolase; eno, enolase; pgi, phosphoglucose isomerase; pgm, phosphoglycerate mutase; tim, triose phosphate isomerase; gapdh, glyceraldehyde 3-phosphate dehydrogenase; pgk, phosphoglycerate kinase; G3P, glyceraldehyde 3-phosphate; PEP, phosphoenolpyruvate; PPP, pentose phosphate pathway. Scale bars represent the average number of substitutions per site

As our analysis drew upon draft genomes (≥ 70% of genome markers present), it is possible that genome incompleteness impacted estimates of presence/absence for metabolic genes. We reasoned that the patchiness metric in particular would be sensitive to this issue, as it is computed (unlike the phylogenetic depth metric) using the number of state transitions of binary characters over the tree. To address this possibility, we undertook a parameter sensitivity analysis that tested the robustness of patchiness scores to genome completeness. We iteratively subsampled the genome set at increasing thresholds of completeness and re-computed trait patchiness over a pruned version of the reference tree (the “Materials and methods” section), observing only modest decreases in patchiness for individual components of the target pathways as genome completeness increased to 95% (Additional file 2, Fig. S4a). Specific glycolytic enzymes showed a similar pattern, with the exception of triose phosphate isomerase (TIM), glyceraldehyde 3-phospate (GADPH), and phosphoglycerate kinase (PGK), which were already essentially universal in CPR bacteria at the lowest completeness threshold (Fig. 3b; Additional file 2, Figure S4ab). Taken together, these results suggest that while genome incompleteness probably impacts calculations of patchiness to a small extent, our observations are mostly due to a biological, not methodological, signal.

Surprisingly, metabolic genes within the same pathway often showed disparate evolutionary profiles—for example, enzymes involved in glycolysis displayed a wide range in depth and patchiness (Fig. 2a). Similar patterns were observed for the nucleotide salvage pathway and non-oxidative pentose phosphate pathway (Fig. 2a). These observations might suggest that evolutionary histories of the component enzymes of these pathways are decoupled; specifically, that traits with high phylogenetic depth and low patchiness are likely ancient and conserved (low loss), whereas those with lower depth and higher patchiness are more likely to have been impacted by loss and/or horizontal gene transfer. To test these hypotheses, we investigated two cases in more detail—first, glycolysis, as an example of a core pathway with a wide range of phylogenetic depth and patchiness among component enzymes, and second, NiFe hydrogenases, an accessory trait with high patchiness and low depth.

### Gene trees for glycolytic enzymes reflect different patterns of gene loss and transfer

We first examined glycolysis, noticing that three enzymes from the central part of the pathway—TIM, GAPDH, and PGK—were found in nearly all CPR bacteria with little to no patchiness (Fig. 3a). With the possible exception of ultra-reduced forms like the Gracilibacteria, which is represented by one complete, curated genome that completely lacks the glycolysis pathway [23], the absence of these enzymes in a very small number of genomes is likely due to missing genomic information. A second group of enzymes, comprised of fructose bisphosphate aldolase (FBA), enolase (ENO), and phosphoglucose isomerase (PGI), was instead generally more patchily distributed among CPR bacteria and missing in some lineages. Phosphoglycerate mutase (PGM), responsible for converting glycerate 1,3-P2 to glycerate 2P in lower glycolysis, fell between the two groups—while present in deeply rooting clades (thus, a high phylogenetic depth), it is absent in several shallow clades of Parcubacteria, possibly because these forms were too divergent to be recovered with the manual HMM threshold. Finally, several enzymes, including glucokinase/hexokinase, phosphofructokinase (PFK), and pyruvate kinase, exhibited profiles that were highly patchy and lower depth among CPR lineages. Notably, these enzymes are thought to catalyze irreversible reactions and thus act as important sites of regulation for metabolic flux [5, 24]. In particular, glucokinase/hexokinase and PFK were found very infrequently in CPR bacteria, though many have the potential to bypass PFK using a metabolic shunt through the non-oxidative pentose phosphate pathway (Fig. 3b) [12]. To confirm this result, we also searched genomes for alternative forms of PFK, finding that while some CPR bacteria encode ROK (repressor, open reading frame, kinase) family proteins (TIGR00744), we could not establish close phylogenetic relationships to family members functioning as putative glucokinases. Likewise, we found no evidence for the alternative versions of ADP-dependent glucokinase/phosphofructokinase employed in the modified glycolytic pathways of some archaea (PF04587) [25].

To further test the impact of genome incompleteness on the apparent patchiness of glycolytic enzymes across the CPR and investigate whether this pattern is unique, we undertook a comparative analysis of other major bacterial phyla. We reasoned that if high patchiness of glycolysis in CPR bacteria is due primarily to genome incompleteness, enzymes from these organisms should have similar patchiness to their counterparts in genomes from other groups with more typical metabolic platforms. On the contrary, if our initial results are indicative of a true biological signal, we would expect enzymes of CPR bacteria to show consistently higher patchiness than observed across other bacterial phyla. We gathered several thousand genomes from metagenomes that were assembled and binned with similar methods to those used to reconstruct genomes of CPR bacteria, corresponding to large phylogenetic groups—Proteobacteria (*n* = 1090), Firmicutes (*n* = 680), and Bacteroidetes (*n* = 578) (the “Materials and methods” section). To ensure comparability of our results, we used the same methodology for genome completeness assessment, metabolic annotation, and analysis of glycolysis as for the CPR. We show that individual glycolysis enzymes from CPR bacteria generally attain the highest patchiness among the lineages examined, particularly for the enzymes at pathway termini (Additional file 2, Fig. S4c). Exceptions include the three glycolysis enzymes that we consider to be a core, essentially universal module across the CPR (TIM, GAPDH, and PGK), and for enolase, where Firmicutes also showed significant patchiness (Additional file 2, Fig. S4c). These findings further confirm that the degree of patchiness observed for glycolytic enzymes in CPR bacteria is robust to issues arising from genome incompleteness and is unusual across major bacterial lineages.

To investigate which specific processes impacted the disparate evolution of glycolytic enzymes in CPR bacteria, we reconstructed single-protein phylogenies and performed gene-species tree reconciliations (the “Materials and methods” section). We reasoned that enzymes whose evolutionary histories were shaped primarily by vertical transfer paired with genomic loss, rather than transfer, would display phylogenetic patterns roughly congruent with our resolved reference species tree, whereas those impacted by horizontal transfer (with either CPR or non-CPR groups) would exhibit incongruent relationships. Gene trees for well-conserved glycolytic capacities like TIM and PGK generally recapitulated phylogenetic groupings at a coarse level (Fig. 3c; Additional file 2, Fig. S5). However, even for these enzymes, inconsistencies with the species tree were present—for example, some TIM sequences from the Microgenomates, Katanobacteria, and Peregrinibacteria clustered with archaeal reference sequences (Fig. 3c). These results were replicated across multiple genomes, and the phylogenetic associations of surrounding ORFs on the same scaffold were verified by BLAST to ensure that the scaffold originated from a CPR organism. Similarly, in the enolase phylogeny, large, monophyletic clusters representing sequences from the Microgenomates and Parcubacteria 1 were resolved; however, other sequences from the Microgenomates and many from Parcubacteria 3 and 4 fell into smaller, fragmented groups that clustered with more distantly related lineages (Fig. 3c). Gene trees for other glycolytic enzymes displayed a range of patterns (Additional file 2, Fig. S5). On the whole, gene-species tree inconsistencies suggest that lateral gene transfer, either between CPR bacteria and other taxa or among different CPR bacteria, has also impacted the evolution of glycolytic enzymes alongside the gene loss apparent from presence/absence profiles (Fig. 3a).

Supporting the possibility of horizontal gene transfer is the observation that multiple distinct enzyme forms underlie the distributions of several glycolytic functions. For example, we recovered unique hits to three individual HMMs representing various versions of PGI—one describing a general, cross-domain version (PF00342), another a bifunctional PGI/phosphomannose isomerase present in some bacteria and archaea (TIGR02128) [26], and, finally, an unrelated cupin-based enzyme originally described from archaea (PF06560) [27, 28]. Interestingly, all three enzymes were scattered across the broad CPR groups, though very few CPR organisms (~ 2% of genomes) encoded more than one version. About 15 genomes, mostly belonging to the Nealsonbacteria, encode only the cupin-related version. These sequences form a sibling clade to those from archaeal reference genomes in the corresponding gene tree (Additional file 2, Fig. S5). Sequences from CPR organisms with highest similarity to archaeal versions were also recovered for PGM (TIGR00306) and for TIM, although in the latter case sequences did not correspond to a separate HMM (Additional file 2, Fig. S5). Similarly, while most CPR bacteria encode a class II FBA enzyme, some, particularly Kaiserbacteria and Woesebacteria, also encode a class I enzyme that functions via a distinct reaction mechanism [29]. Finally, in gene tree reconstructions for the class II aldolase, sequences from CPR bacteria do not appear to be monophyletic, with small subgroups dispersed among sequences from other bacteria. Taken together, these results indicate that enzymes of multiple evolutionary origins underlie the distributions of core carbon metabolism, and support the idea that their distributions have been shaped by episodes of lateral gene transfer, potentially from non-CPR bacteria or archaea.

### CPR bacteria encode phylogenetically distinct forms of NiFe hydrogenases with variable genomic context

**Fig. 4:**
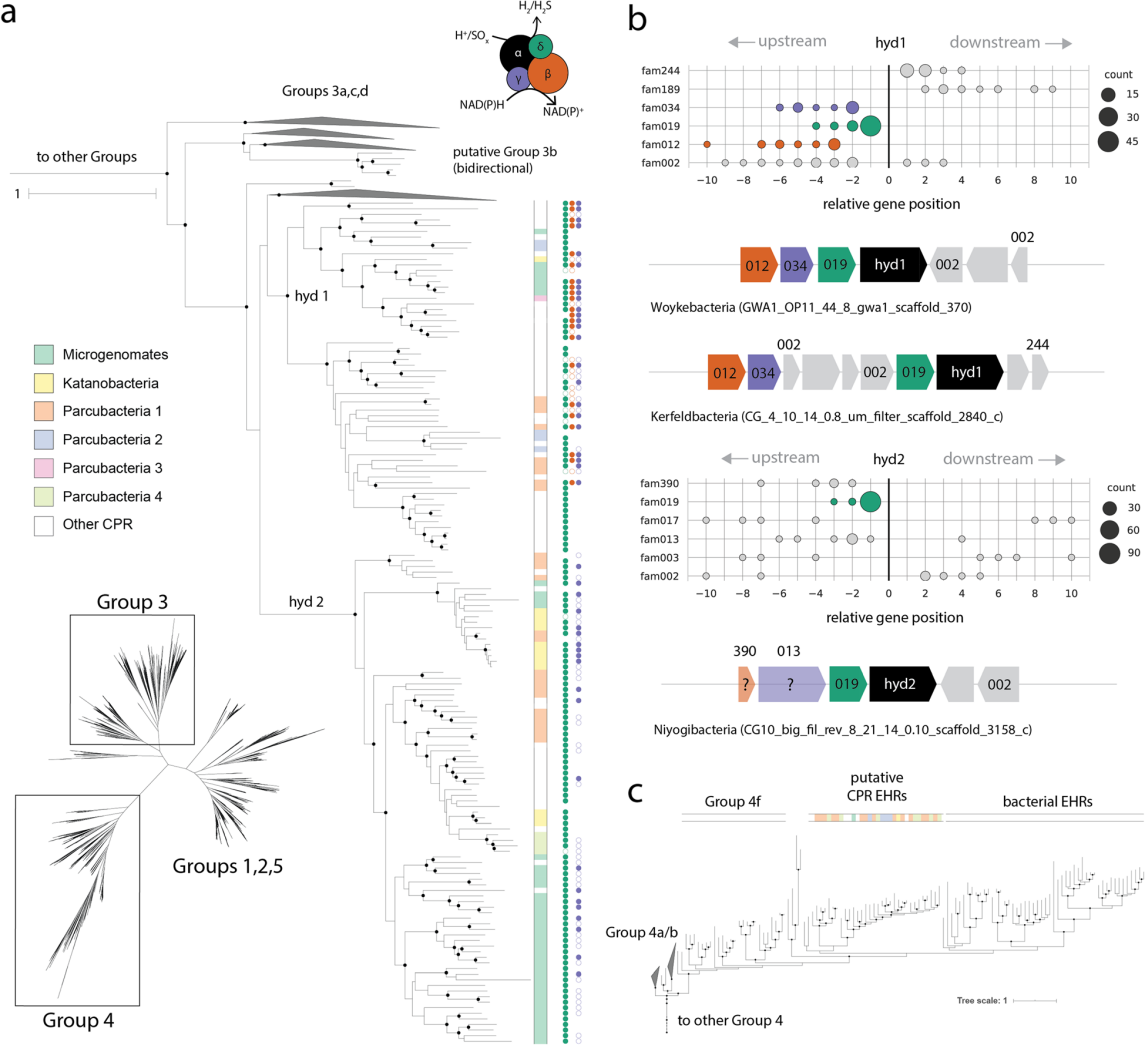
NiFe hydrogenase enzymes encoded by CPR bacteria. **a** Inset of the unrooted large subunit hydrogenase tree showing putative Group 3b hydrogenases across the CPR, along with the presence/absence of HMM hits corresponding to other subunits. **b** Genomic context for hydrogenase gene clusters, where position 0 corresponds to the location of the ORF encoding the large subunit of the NiFe hydrogenase. Only protein families on the same strand as the large subunit are represented in the plots, whereas genome diagrams below the charts include all proximal families regardless of strand orientation. **c** Inset of large subunit tree within Group 4 hydrogenases. Ehr, energy-converting hydrogenase-related complexes. Scale bars represent the average number of substitutions per site

We next investigated the impact of lateral transfer on metabolisms sparsely distributed across the CPR, focusing on the NiFe hydrogenases as a case study because of their possible role in hydrogen economy and/or electron flux [5, 18]*.* Most sequences from CPR bacteria were previously reported to fall within the Group 3b hydrogenases, cytoplasmic enzymes that may catalyze the reversible oxidation of H_2_ coupled to regeneration of NADPH or reduction of polysulfide when available [30, 31]. Here, a revised gene tree that broadly samples the CPR reveals the presence of two subclades, which we term *hyd1* and *hyd2*, forming a larger clade of Group 3b hydrogenase from CPR organisms (Fig. 4a). Both groups are related to, but distinct from, Group 3b versions in other bacteria and archaea, particularly *hyd2*, which is separated from its sibling clades by a relatively long branch (Fig. 4a).

Biochemically characterized Group 3b NiFe hydrogenases are known to be tetrameric enzymes [32]. To examine whether subunit associations were consistent across hydrogenase classes, we probed the genomic context of the large subunits from CPR bacteria using a paired HMM-protein clustering approach (the “Materials and methods” section). Intriguingly, while both enzyme types were generally associated with the small subunit hydrogenase (fam019) in addition to the catalytic subunit, only *hyd1* co-located with genes encoding protein families resembling the two other subunits involved in NAD(P)^+^-binding (gamma, fam034) and electron transfer (beta, fam012) (Fig. 4a). HMM searches revealed that these subunits also have homology to anaerobic sulfide reductase A and B, suggesting that the entire complex could be involved in sulfur metabolism through the reduction of reduced sulfur compounds like polysulfide [32, 33]. However, in some cases, the gamma and beta subunits were not immediately upstream from the gene encoding the small subunit (Fig. 4b), and, in others, were not detected at all (Fig. 4a). This inconsistency might be due to genome incompleteness or lineage-specific losses within the *hyd1* clade.

Although genomes with *hyd2* also encoded the small subunit protein (fam019), the sequences were consistently truncated (mean 164 amino acids) relative to those associated with *hyd1* and non-CPR bacteria (mean 250 amino acids) (Additional file 2, Fig. S6a) [34]. Both forms also shared fam002 in their genomic context, some members of which displayed homology to the hydrogenase-associated chaperone *hypC*. Outside these families, immediate genomic context differed for *hyd2*: while an HMM search recovered sequences with the NAD-binding motif (gamma subunit) in the vicinity of some *hyd2*, protein clustering showed that these proteins were neither proximal to nor on the same strand as the catalytic subunit (Fig. 4ab). However, some members of fam013 that were in the genomic context of *hyd2* apparently possessed an NAD(P)-binding domain situated within a larger FAD-binding domain (PF07992). Similarly, while HMM searches did not recover evidence for a putative beta subunit near *hyd2*, we found one protein family (fam390) in proximity to a subset of *hyd2* that contained one of two iron-sulfur-binding domains. These domains were distinct from those associated with the putative beta subunit near *hyd1.* Ultimately, it is unclear whether *hyd2* consistently possesses (or lacks) the gamma and beta subunits, and thus, its function remains uncertain.

Intriguingly, both *hyd1* and *hyd2* were dispersed across many lineages of the CPR, and some lineages contained both subtypes in closely related but distinct genomes (Fig. 4a). For example, genomes from the Roizmanbacteria, which harbored the largest total number of Group 3b-related NiFe hydrogenases (*n = 15*), individually contained either *hyd1* or *hyd2* sequences. Mapping of genome taxonomy onto the 3b-related hydrogenase tree confirmed incongruencies with the CPR species tree (Fig. 4a). A similar pattern was observed for sequences from CPR bacteria that fell within a subclade of Group 4 references representing energy-converting hydrogenase-related complexes (Ehr). Notably, the sequences from CPR bacteria were monophyletic and clustered separately from other Ehr proteins, although they also lacked the cysteine residues that bind the metal cofactors in other Group 4 enzymes (Additional file 2, Fig. S6b). This observation suggests that Ehr proteins from CPR organisms likely cannot interact with H_2_.

## Discussion

Initially described as a radiation of phylum-level clades based on analyses of 16S rRNA divergence [1], the CPR was initially suggested to comprise at least 15% of bacterial phylum-level groups [1]. Subsequent analyses have suggested that its scale potentially matches that of all other bacterial diversity [10]. Attempts to adjust for lineage-specific evolutionary rates have suggested the collapse of the CPR into a single phylum [9], but more recent analyses with balanced taxonomic sampling continue to depict it as a large component part of bacterial diversity [11]. Here, we combined new and previously reported genomes to construct a robust reference phylogeny for the CPR using two unlinked, concatenated marker sets (Fig. 1a, Additional file 2, Fig. S2). The reconstructed trees are generally consistent with, and more clearly define, the topology originally described for the CPR [1], although definitive resolution of some deep nodes, particularly those connecting divergent groups like the Saccharibacteria, Gracilibacteria, and Absconditabacteria (SR1), remain elusive, possibly due to undersampling of the latter two lineages. Both gene trees support the presence of several monophyletic subgroups within the Parcubacteria, motivating subdivision of this large clade into smaller, taxonomically relevant units.

Here, we evaluated metabolic platforms across the CPR by mapping genomically encoded functions onto the reference tree. Analysis of metabolic capacity among CPR organisms presents several challenges, primarily due to the fact that homologs of metabolic genes are often highly divergent compared to known reference sequences. Our custom approach for determining suitable cutoffs for HMMs indicates that manual threshold curation is important when proteins are only distantly related to biochemically characterized versions (Additional file 2, Figs. S1 and S3). We found that metabolic platforms for CPR lineages only partially mirror phylogenetic relationships (Fig. 1a, c), at least for the subset of metabolic traits examined here. In other words, phylogenetically distant lineages often possessed combinations of metabolic capacities that were more similar to each other than to those of more closely related clades (Fig. 1c). Thus, we hypothesize that diverse lineages within the CPR may have converged upon similar metabolic platforms, potentially via combinations of lateral gene transfer and gene loss of genes involved in the same function(s). This finding is intriguing, given that overall protein presence/absence patterns in both CPR and other bacteria generally recapitulate phylogenetic relationships when entire proteomes are considered [15]. To account for this difference, we infer that other protein families not included in the current study must show patterns of presence/absence that are generally congruent with the CPR species tree.

Exploration of patterns of gene distribution revealed that patchiness and phylogenetic depth varied for the selected metabolisms and even for enzymes in the same pathway (Fig. 2). This finding was validated by additional analyses of how trait patchiness varied with increasing completeness of the underlying genomes, and, for glycolysis in particular, by a comparison to other major bacterial groups. Based on the combination of these analyses, we conclude that incompleteness of genomes from metagenomes used in this study only minimally alters the relative relationships between traits when examining depth and patchiness and that the unusual patterns observed for CPR organisms are indeed atypical. Similarly, while mis-binning can also complicate any analysis that relies upon metagenome-derived genomes, the similarity of findings for multiple closely related genomes indicates that it likely does not greatly obscure the major patterns presented here. While the increased availability of complete genomes will best help to further clarify the patterns explored in this study, our general approach to testing the robustness of signal as a function of genome completeness might serve as a valuable way to augment future analyses of gene content in other lineages as well.

We then used gene-species tree reconciliation to validate the prediction that proteins with variable “evolutionary profiles” might have been shaped by different combinations of lateral transfer and vertical inheritance. For a subset of core carbon metabolism, here represented by glycolysis, gene trees were roughly congruent with the reconstructed organismal phylogeny, suggesting that vertical inheritance has primarily shaped distributions of these enzymes (Fig. 3). However, the discovery of a divergent subclade of TIM from CPR bacteria that is more closely related to archaeal versions than bacterial ones provides clear evidence of lateral transfer even for the most widely distributed glycolytic enzymes. Interestingly, two enzymes involved in the early steps of the glycolytic pathway (hexokinase/glucokinase and phosphofructokinase) were notably absent in nearly all lineages. Where present, they were likely acquired by lateral gene transfer, potentially following ancestral loss. These sequences separate from those of other bacteria, obscuring the source and suggesting that transfers of phosphofructokinase and hexokinase to CPR were also ancient. In contrast, enolase and pyruvate kinase, the last two steps of the pathway, are only somewhat widespread and show relatively low phylogenetic congruence. This pattern may reflect a mixture of genomic loss in addition to lateral transfer among unrelated CPR organisms.

In archaea, glycolysis is known to be modified in a number of ways, including metabolic shunting [35] and rewiring of steps through novel enzymes [36, 37]. These observations have led to suggestions that evolutionary “tinkering” has shaped glycolysis at least in some archaeal lineages [38]. Paralleling this, we found that several glycolytic steps in CPR bacteria are apparently carried out by different enzyme forms, and, in some cases, by types that are traditionally associated with archaea. This was particularly striking in the case of PGI, which converts glucose 6-P to fructose 6-P, where three different enzyme forms accounted for the wide distribution of the function (Fig. 3a). Acquisition of variant enzymes may have preceded loss of the ancestral enzyme or occurred afterwards, complementing a loss in function. Overall, our findings suggest that glycolysis among CPR organisms is partly an evolutionary mosaic, as described in at least one eukaryotic organism (the flagellate *Trimastix pyriformis*) [39], and, further, that gene loss and acquisition may have remodeled their glycolytic pathways over time.

Given the patchy distribution of enzymes involved in upper glycolysis, carbon flux through this portion of the pathway remains unclear. CPR bacteria without glucokinase/hexokinase (hex) or PGI might rely on the uptake of glycolytic intermediates, like fructose 6P or fructose 1,6-P2 from associated cells or released by cell lysis. These compounds could be shunted through the pentose phosphate pathway to bypass the largely absent phosphofructokinase and into the conserved central module of glycolysis (Fig. 3a) [5]. Alternatively, near universal conservation of TIM and GAPDH across the CPR suggests that either glycerone or G3P could also be important points of input for carbon flow in these organisms. Consistent with this is the fact that CPR organisms encoding FormIII-related RuBisCO are predicted to introduce G3P to central/lower glycolysis as a product of their predicted nucleotide salvage pathway [21, 40]. The subset of CPR organisms that encode both hexokinase and PGI, on the other hand, could potentially perform a more diverse set of transformations, utilizing glucose precursors taken up from the environment or host. As for lower glycolysis, the observed patchiness in distributions of PGM, enolase, and pyruvate kinase suggests alternative fates for intermediates produced after the step catalyzed by PGK (Fig. 3a). In the absence of pyruvate kinase, which was found only in about a third of genomes here, CPR could use phosphoenolpyruvate (PEP) synthetase (PEPS) to instead interconvert PEP and pyruvate or instead generate oxaloacetate [41]. Of course, with the data presented here, we cannot rule out the possibility that novel, divergent enzymes undetected by our HMM approach functionally substitute for those with patchy or nearly absent distributions among CPR lineages. However, we found no evidence for the presence of archaeal PFK/glucokinase nor strong support for functioning of CPR ROK family proteins as putative glucokinases. Additionally, CPR bacteria are not currently known to employ alternative pathways like the Entner-Doudoroff pathway, as some other bacteria that lack PFK [42]. Future work subjecting CPR organisms in culture/co-culture to carbon flux analysis should help to validate genomic predictions and shed light on the metabolic configurations utilized in vivo*.*

Our second case study investigated the evolutionary history of specialized metabolism in CPR bacteria, focusing on Group 4 and 3b NiFe hydrogenases (Fig. 4). These genes, like those putatively involved in nitrite reduction, electron transport, and AMP metabolism [16, 20, 43], are sparsely distributed across the CPR and were likely subjected to lateral gene transfer. Notably, we report phylogenetic and genomic evidence for distinct monophyletic clades of Group 3b hydrogenases that are specific to the CPR. This suggests that transfer events were ancient or that these hydrogenase sequences evolved very rapidly. The variable genomic contexts of the 3b-related *hyd1* and *hyd2* suggest at least two evolutionary scenarios: that individual, ancient transfers from non-CPR microorganisms occurred with the associated proteins intact, or that CPR bacteria encoding *hyd2* acquired only the large and small subunit and currently support function with unknown genes. The scattered distribution of both forms, phylogenetically incongruent with the CPR species tree, further suggests that intra-CPR exchange and/or loss also occurred over time. Similarly, we hypothesize that other sparsely distributed protein families among the CPR, like pyruvate:ferredoxin oxidoreductase, cytochrome oxidase, and nirK (nitrite metabolism), may also be the result of lateral transfer followed by further evolution within the CPR. The acquisition of cytochrome oxidase by some Saccharibacteria is presumably an adaptation to aerobic or microaerophilic environments [5, 12, 44].

In contemplating modes of evolution of CPR bacteria, it is important to consider the processes of gene gain and loss in the context of the largely symbiotic lifestyles of these organisms. The dynamic evolution of glycolysis might reflect reduced selection for complete pathways due to metabolic opportunities provided by the host, constraints which probably changed over time. Further, acquisition of new capacities via lateral transfer could have opened new niches, potentially including a change in or adaptation to new hosts in different environments. However, the observation that sequences from CPR bacteria coding for rarer functions are often distinct from those of other bacteria suggests that these transfers probably occurred relatively early in the history of the radiation, or evolved rapidly once acquired. Distantly related lineages within CPR may have independently undergone loss or gain of the same set of protein families, leading to similarly reduced metabolic platforms over time. These evolutionary constraints may be unique compared to those shaping minimal metabolism in other non-CPR bacterial groups with reduced genomes, like endosymbionts of insects. In contrast to these relatively recently evolved (linked to the appearance of eukaryotic hosts) associations that probably involve irreversible genome reduction trajectories [45], the potential for CPR organisms to associate with other bacteria raises the possibility of long-established symbioses in which gene sets remain in flux. The resulting pattern of “diversity within sparsity” appears to be characteristic of the CPR.

## Materials and methods

### Genome collection and construction of phylogenetic marker sets

We compiled a large set of genomes from metagenomes from CPR bacteria from several previous studies of various environments. We also binned an additional set of genomes from metagenomes previously generated from sediment from Rifle, Colorado [4], groundwater from Crystal Geyser [46, 47], a cyanobacterial mat from the Eel River network in northern California [48], groundwater from a cold sulfide spring in Alum Rock, CA, and human saliva. Binning methods and taxonomic assignment followed Anantharaman et al. [4]. The total set was initially filtered for genomes that had been manually curated by any method to reduce the occurrence of mis-binning, yielding a starting set of approximately 3800 genomes. We next computed contamination and completeness for all genomes using a set of 43 marker genes sensitive to described lineage-specific losses in the CPR [1, 4] using the custom workflow in checkm [49]. Results were then used to secondarily filter the genome set to those with ≥ 70% of the 43 marker genes present and ≤ 10% of marker genes duplicated. The resulting ~ 2300 genomes were de-replicated at 95% ANI using dRep (*-sa 0.95 -comp 70 -con 10*) [50], yielding a set of 991 non-redundant genomes used for downstream analysis. These genomes along with their associated information, including accession numbers/links, are listed in Additional file 1, Table S1.

We re-predicted genes for each genome using Prodigal (“single” mode) [51], adjusting the translation table (*-g 25*) for CPR lineages (Gracilibacteria and Absconditabacteria) known to utilize an alternative genetic code. Next, we assembled two sets of HMMs, representing the 16 syntenic ribosomal proteins (rp16) and, separately, the two subunits of RNA polymerase (RNAp), from the TIGRFAMs and Pfams databases and ran each against predicted proteins using HMMER v3.1b2 (http://hmmer.org). To maximize extracted phylogenetic information, including partial genes with robust homology to the marker genes, we set custom thresholds for each HMM using trees generated from all significant (*e* < 0.05) hits to a given HMM (aligned using MAFFT, tree inference with FastTreeMP) [52, 53]. Thresholds were usually set at the highest bitscore attained by proteins outside the clade of interest (Additional file 2, Fig. S1), which were verified with BLASTp. HMM results and thresholds were visualized by in bitscore vs. *e* value plots (Additional file 2, Figure S1ab). Phylogenetic analysis of HMM hits revealed that many proteins below model-specific thresholds were legitimate, often partial hits to the targeted HMM (Additional file 2, Fig. S1b).

Next, we curated phylogenetic marker sets for both rp16 and RNAp by addressing marker genes present in multiple copies in a given genomic bin. Multi-copy genes can result from remnant contamination after filtering, ambiguous bases in assembly leading to erroneous gene prediction [49], or legitimate biological features. We first identified marker genes fragmented by errors in gene prediction by searching for contiguous, above-threshold hits to the same HMM on the same assembled contig. This issue was particularly prevalent for rpoB and rpoB’, possibly due to repetitive regions in that gene impacting accurate assembly. For upstream fragments, we removed protein residues after stretches of ambiguous sequence to avoid introducing mis-translated bases into the alignment stage while maximizing phylogenetic information. If additional stretches of ambiguous sequence were present in downstream fragments, we removed them. Finally, we built a corrected, non-redundant marker set for each genome by selecting the 16 ribosomal proteins and, separately, 2 RNA polymerase subunits that firstly maximized the number of marker genes on the same stretch of assembled DNA and, secondarily, maximized the combined length of encoded marker genes.

### Species tree inference, curation, and analysis

Results for each marker gene in the rp16 and RNAp sets were individually aligned with MAFFT [52] and subsequently trimmed for phylogenetically informative regions using BMGE (-m BLOSUM30) [54]. Gene trees for each marker were then constructed using IQTREE’s model selection and inference (*-m TEST -nt AUTO -st AA*) and manually inspected for major incongruencies.

In preparation for creating a concatenated alignment for each marker set, we next extracted corresponding rp16 and RNAp marker sets for a diverse bacterial outgroup consisting of ~ 170 bacterial genomes from GenBank sampled evenly across characterized taxonomic divisions. We then merged the outgroup dataset with the existing marker gene sets, individually aligning hits for each marker gene and trimming them as described above. We then concatenated individual protein alignments, retaining only those with both RNAp subunits and at least 8 of 16 syntenic ribosomal proteins. Maximum-likelihood trees were inferred for both the concatenated rp16 (1427 AA) and RNAp (1652 AA) sets using ultrafast bootstrap and IQTREE’s extended FreeRate model selection (*-m MFP -st AA -bb 1500*) [55–57], given the importance of allowing for site pattern heterogeneity in concatenated alignments [58]. FASTA-formatted files for the masked alignment and newick-formatted trees for both rp16 and RNAp datasets are available in Additional file 4.

We next identified phylogenetic outliers in the resolved maximum-likelihood topologies by searching for genomes that did not form a monophyletic clade with other organisms of the same taxonomy. These genomes, potentially due to mixed phylogenetic signal or undersampling, were retained only if they were assigned to a previously described novel lineage, or formed a conserved, uncharacterized clade with > 1 member in both rp16 and RNAp trees. Genomes that did not fit these criteria were pruned. Concatenated trees were then re-inferred with the modified genome set. Where possible, we manually curated taxonomic assignments for genomes that clearly resolved within monophyletic clades of different taxonomic classification in both the rp16 and RNAp trees. Finally, we assessed broad-scale phylogenetic patterning within the CPR by examining the distribution of ribosomal proteins L1 and L9 employing the same HMM-based approach as described above.

### Metabolic annotation, analysis, and gene tree inference

To probe metabolism within CPR bacteria, we assembled a broad set of HMMs from TIGRFAMs (tigrfams.jcvi.org/cgi-bin/Listing.cgi), Pfam (pfam.xfam.org), and a previous publication [4] representing metabolisms relevant for biogeochemical cycling and energy production in this clade [5, 12, 18] (Additional file 3, Table S2). We interrogated protein sequences from each genome with the HMM set using HMMER and set custom bitscore thresholds as described above to ensure that divergent but functionally valid proteins were retained. Model-specific thresholds were often much higher than maximum bitscores of hits, even in cases where we were able to assign putative function to relatively high scoring clusters through BLAST and phylogenetic analyses. In a few cases (PRPP, PEP synthase, PGI, ROK family), we secondarily annotated HMM-protein hits with additional Pfam domains or manually inspected placement within a reference tree to guide setting of accurate manual cutoffs. These additional domain HMMs and all custom thresholds are specific to this dataset and are listed in Additional file 3, Table S2. If a protein had multiple above-threshold hits to a set of HMMs, we selected the HMM with the highest bitscore. We additionally selected the highest-scoring HMM hit within a genome bin for each HMM to generate a final set of metabolic markers for downstream analysis.

We next analyzed distributions of metabolic capacities in two ways: first, we created a presence/absence matrix for all metabolisms with at least one hit among the genome set, combining profiles for HMMs representing the same function (e.g., PGI, FBA, RuBisCO) into a single merged category. We then filtered the matrix to include only lineages with eight or more genomes and traits that were detected at least three times over all genomes. Finally, we averaged presence/absence across lineages, generating a frequency at which that trait was present among genomes of a particular taxonomy. We then used this information to generate a Bray-Curtis distance matrix using the ecopy package in Python. Finally, we performed a principal coordinates analysis using scikit-bio learn and plotted the resulting axes to examine clustering and variation within and among metabolic platforms of CPR bacteria. Second, we measured phylogenetic conservation and patchiness over the rp16 tree using the consenTRAIT algorithm (*Npermutations = 1000, count_singletons = F, min_fraction = 0.90*) [59] as implemented in the R package *castor* and consistency index (CI) as implemented in the R package *phangorn* and proposed in [22] (*sitewise = T*). We integrated these two metrics to generate an “evolutionary profile” for each gene.

To assess how patchiness of given metabolisms varied with genome completeness, we subsampled the genome set iteratively at increasing thresholds from 70% through 95% and, for each iteration, pruned down the existing rp16 reference tree to include only those genomes. We then re-computed patchiness for each trait as done previously. For the comparative analysis of patchiness among glycolytic enzymes, we gathered all non-CPR bacterial genomes from three major studies of groundwater microbial communities from which the majority of CPR bacterial genomes were assembled [4, 46, 47]. To increase our phylogenetic sampling, we combined this set with additional genomes-from-metagenomes from a large-scale study of multiple environments that used similar methods [2] and selected three major lineages with adequate size to use for downstream analysis (Proteobacteria, Firmicutes, and Bacteroidetes). We calculated completeness and contamination for the non-CPR genomes using the same set of 43 markers as before and de-replicated them at 95% ANI with dRep, using the calculated completeness and contamination to again filter at 70% completeness and 10% contamination. We next extracted the rp16 phylogenetic markers using a similar approach (though, for simplicity, HMMs were thresholded using model-specific noise cutoffs) and processed as before. Next, a random subsample of 50 CPR bacterial genomes was taken and their phylogenetic markers were separately concatenated with those of each non-CPR lineage. Alignment, alignment trimming, and tree building were performed as previously for each set of sequences. For each of the three non-CPR groups, HMMs corresponding to glycolytic enzymes were run against predicted proteins and manually re-thresholded. Finally, genome sets for each lineage were subsampled at increasing completeness thresholds as for CPR bacteria, and patchiness was computed for each glycolysis enzyme over each tree as above. Results were combined with those obtained for CPR organisms and visualized.

To build reference protein sets for the metabolic genes of interest, we queried proteins from the set of ~ 170 bacterial reference genomes with same HMMs described above and applied the model-specific noise cutoff (for Pfam or TIGRFAMs HMMs) or the published cutoff (for custom HMMs). These proteins were then concatenated with the corresponding above-threshold hits from the CPR bacterial genomes and aligned as described above with MAFFT. Additionally, for four HMMs corresponding to glycolytic functions (PF06560, TIGR02128, TIGR00306, TIGR00419), we also queried a set of proteins from ~ 300 archaeal reference genomes assembled in a similar fashion to the bacterial reference set. Resulting protein hits were concatenated with the bacterial sequences. For all single-gene alignments, columns with 95% or more gaps were trimmed using Geneious. Maximum-likelihood gene trees were then inferred using IQTREE with the following parameters: *-m TEST -st AA -bb 1500.* Trees were rooted on the largest monophyletic group of reference sequences present in the topology; if multiple monophyletic groups of reference sequences were present, trees were rooted at the midpoint.

To generate a gene tree for the NiFe hydrogenases, we assembled a comprehensive reference set of large subunit sequences from several published sources [60–62], de-replicated them at 95% amino acid identity using *usearch --cluster_fast*, and concatenated the resulting centroids with large subunit sequences recovered from CPR bacteria. Sequences were aligned, alignments were trimmed, and the gene tree was inferred as described above for other metabolic genes. The trimmed alignment is available in Additional file 4. We next manually identified sequences within the immediate genomic context of 3b-related catalytic subunits that also scored highly against HMMs for anaerobic sulfite reductase A/B, as described previously for subunits in the Group 3b hydrogenases of *Pyrococcus furiosus* [32, 33] and searched them for conserved domains in phmmer (https://www.ebi.ac.uk/Tools/hmmer/search/phmmer). We identified one iron-sulfur cluster and one NAD-binding domain that were conserved among these proximal proteins (Additional file 3, Table S2), and then queried all proteins from CPR bacteria with these HMMs to identify putative 3b-related subunits across the entire genome set. We performed the same search for an additional Pfam domain associated with the 3b-hydrogenase small subunit (Additional file 3, Table S2). For all three HMMs, manual thresholds were set using the paired visualization-phylogenetic approach described above. Finally, the presence/absence of putative subunits was mapped onto the resolved tree of large subunit sequences to examine patterns of association with phylogenetic clades of 3b-related hydrogenase using iTol [63].

For the genomic context analysis of 3b-related forms, we gathered protein sequences within a 20 ORF radius (or less, if the scaffold ended) in both directions of the identified large subunits. Each ORF was assigned a genomic position relative to the large subunit (position 0). All recovered proteins were concatenated into a single file and passed through a two-part, de novo protein clustering pipeline recently applied to CPR genomes, in which proteins are first clustered into “subfamilies” and highly similar/overlapping subfamilies are merged using and HMM-HMM comparison approach (--coverage 0.50) [15] (https://github.com/raphael-upmc/proteinClusteringPipeline). Recovered protein families were compared with subunit HMM results and linked if the majority of proteins within the family had above-threshold hits to a given HMM. An alignment and gene tree for those proteins labeled as the small subunit hydrogenase (fam019) were made as described above.

Finally, counts for genes encoding the recovered families were plotted as a function of their relative position to the focal catalytic subunit of the hydrogenase across all CPR bacterial genomes. This was performed only if there were instances of the genes on the same strand (as predicted by Prodigal) as the large subunit hydrogenase. The relative positions of genes were multiplied by their strand orientation such that a negative position would signify being “upstream” of the focal catalytic subunit, whereas a positive position would signify being “downstream.” Positions were also adjusted in several cases where the focal subunit was split into multiple consecutive fragments, possibly due to local assembly errors.

## Supplementary information

**Additional file 1: Table S1.** Characteristics of genomes used in this study. (TSV 127 kb)

**Additional file 2: Figure S1.** Visual and phylogenetic approach to setting sensitive manual thresholds for phylogenetic markers. HMM rank vs. bitscore/e-value plot for **a)** ribosomal protein S3 (TIGR01009) and **b)** RNA polymerase, subunit beta (TIGR02013). **c)** Molecular phylogeny for significant (e > 0.05) TIGR02013 hits onto which HMM scores from **b)** are mapped. **Figure S2.** Consistent tree topology for the CPR recovered individually by a concatenation of **a)** 16 ribosomal proteins and **b)** B and B′ subunits of RNA polymerase. Clade shading corresponds to that in Fig. 1a. Scale bars represent the average number of substitutions per site. Ultrafast bootstrap support is indicated by the number attached to each tree node. **Figure S3.** Visual and phylogenetic approach to setting sensitive manual thresholds for metabolic genes of interest. HMM rank vs. bitscore/e-value plot for **a)** fructose 1,6-bisphosphatase (PF00316) and **b)** triose phosphate isomerase (TIGR00419). Molecular phylogeny for significant (e > 0.05) hits to **c)** PF00316 and **d)** TIGR00419 onto which HMM scores are mapped. **Figure S4.** Impact of genome completeness on patchiness for **a)** all enzymes within the major pathways in CPR bacteria examined in this study and **b)** individual enzymes within CPR glycolysis. Part **c)** shows the patchiness of each individual glycolytic enzyme as a function of genome completeness for four major lineages, including the CPR. PPP=Pentose Phosphate Pathway. dh = dehydrogenase. **Figure S5.** Maximum-likelihood gene trees for glycolytic enzymes in CPR bacteria. Different HMMs representing the same functions are grouped together by boxes. Scale bars represent the average number of substitutions per site. Black dots indicate tree nodes with > = 95% ultrafast bootstrap support. **Figure S6. a)** Maximum-likelihood gene tree for 3b-related NiFe hydrogenase small subunit (SSU) (fam019) with trimmed protein alignment for SSU sequences. Scale bar represents the average number of substitutions per site. Black dots indicate tree nodes with > = 95% ultrafast bootstrap support. **b)** Partial alignment of the L1 and L2 regions of putative Group 4-related NiFe hydrogenases. Ehr  = energy-converting hydrogenases-related complexes. Red asterisk indicates cysteine residues associated with metal cofactor binding. **N.B.** for visual clarity, only a subset of sequences and sites are shown.

**Additional file 3: Table S2.** Description of metabolic HMMs and thresholds used in this study. (TSV 22 kb)

**Additional file 4.** Trimmed alignments and inferred maximum-likelihood trees for the concatenated rp16 set (rp16_concat.mafft and rp16_concat.treefile), concatenated RNAp set (rpol_concat.mafft and rpol_concat.treefile), and NiFe hydrogenase large subunit (hyd.mafft and hyd.treefile).

## Data Availability

The genomes analyzed in this study are available either from the NCBI GenBank/Biosample repositories (previously published genomes) or from Zenodo (genomes newly assembled in this study). All accessions/links are listed in Additional file 1, Table S1. All genomes, as well as intermediate data files, including sequence files, and custom code used for the described analyses are available as a GitHub repository: https://github.com/alexanderjaffe/cpr-phylo-metab [64].
