# Supplementary material for: The rise of diversity in metabolic platforms across the Candidate Phyla Radiation
Source: BMC Biol. 2020 Jun 19;18:69. doi: 10.1186/s12915-020-00804-5 (PMC7304191; doi:10.1186/s12915-020-00804-5)
Supplement: Supplementary file 2 — Additional file 2: Figure S1. Visual and phylogenetic approach to setting sensitive manual thresholds for phylogenetic markers. HMM rank vs. bitscore/e-value plot for a) ribosomal protein S3 (TIGR01009) and b) RNA polymerase, subunit beta (TIGR02013). c) Molecular phylogeny for significant (e > 0.05) TIGR02013 hits onto which HMM scores from b) are mapped. Figure S2. Consistent tree topology for the CPR recovered individually by a concatenation of a) 16 ribosomal proteins and b) B and B′ subunits of RNA polymerase. Clade shading corresponds to that in Fig. 1a. Scale bars represent the average number of substitutions per site. Ultrafast bootstrap support is indicated by the number attached to each tree node. Figure S3. Visual and phylogenetic approach to setting sensitive manual thresholds for metabolic genes of interest. HMM rank vs. bitscore/e-value plot for a) fructose 1,6-bisphosphatase (PF00316) and b) triose phosphate isomerase (TIGR00419). Molecular phylogeny for significant (e > 0.05) hits to c) PF00316 and d) TIGR00419 onto which HMM scores are mapped. Figure S4. Impact of genome completeness on patchiness for a) all enzymes within the major pathways in CPR bacteria examined in this study and b) individual enzymes within CPR glycolysis. Part c) shows the patchiness of each individual glycolytic enzyme as a function of genome completeness for four major lineages, including the CPR. PPP=Pentose Phosphate Pathway. dh = dehydrogenase. Figure S5. Maximum-likelihood gene trees for glycolytic enzymes in CPR bacteria. Different HMMs representing the same functions are grouped together by boxes. Scale bars represent the average number of substitutions per site. Black dots indicate tree nodes with > = 95% ultrafast bootstrap support. Figure S6. a) Maximum-likelihood gene tree for 3b-related NiFe hydrogenase small subunit (SSU) (fam019) with trimmed protein alignment for SSU sequences. Scale bar represents the average number of substitutions per site. Black dots indica [file 12915_2020_804_MOESM2_ESM.pdf]

ADDITIONAL FILE 2: FIGURES S1-6

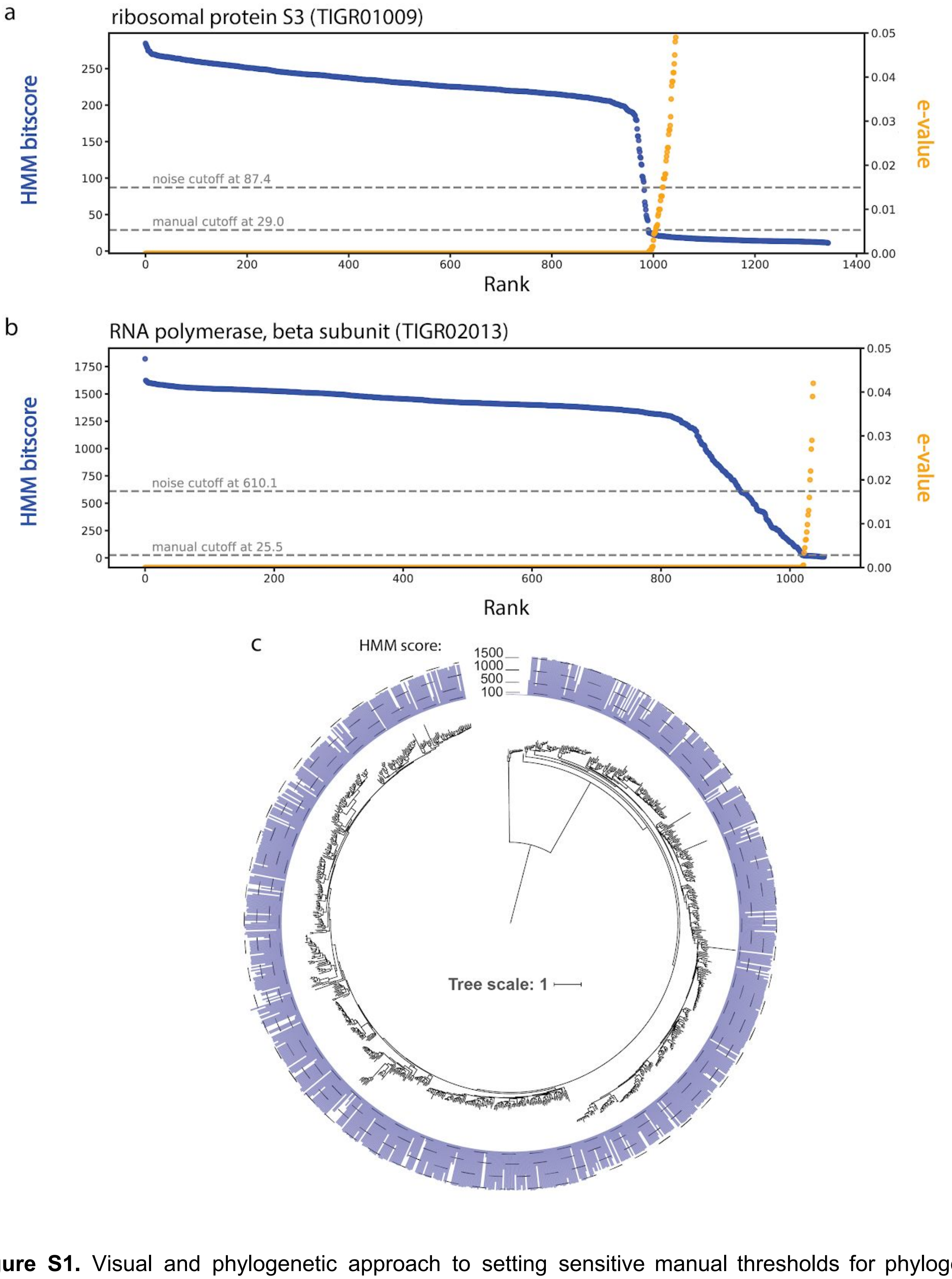

Figure S1. Visual and phylogenetic approach to setting sensitive manual thresholds for phylogenetic markers. HMM rank vs. bitscore/e-value plot for **a)** ribosomal protein S3 (TIGR01009) and **b)** RNA polymerase, subunit beta (TIGR02013). **c)** molecular phylogeny for significant (e > 0.05) TIGR02013 hits onto which HMM scores from **b)** are mapped.

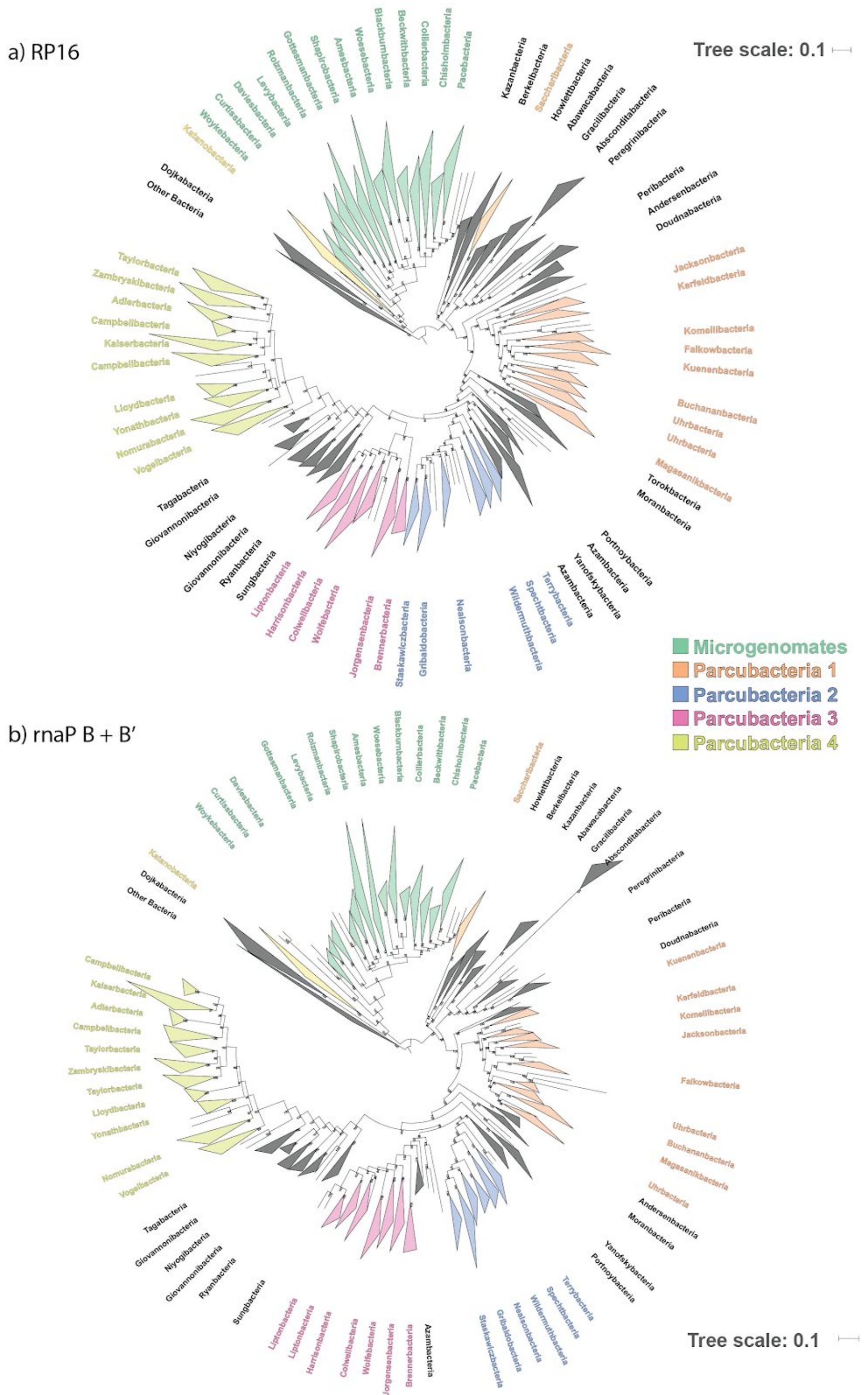

Figure S2. Consistent tree topology for the CPR recovered individually by a concatenation of **a)** 16 ribosomal proteins and **b)** B and B' subunits of RNA polymerase. Clade shading corresponds to that in Fig 1a. Scale bars represent the average number of substitutions per site. Ultrafast bootstrap support is indicated by the number attached to each tree node.

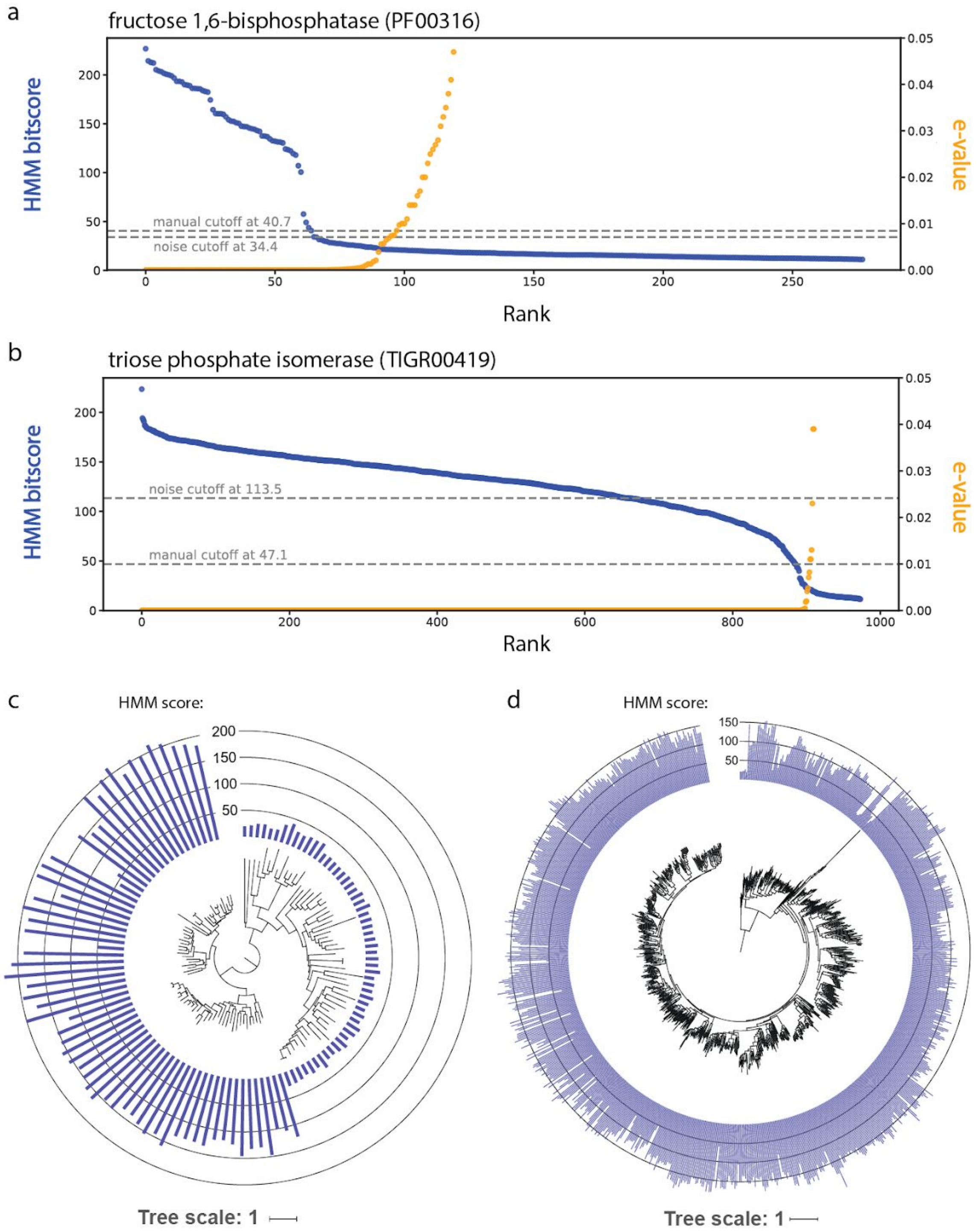

Figure S3. Visual and phylogenetic approach to setting sensitive manual thresholds for metabolic genes of interest. HMM rank vs. bitscore/e-value plot for **a)** fructose 1,6-bisphosphatase (PF00316) and **b)** triose phosphate isomerase (TIGR00419). Molecular phylogeny for significant (e > 0.05) hits to **c)** PF00316 and **d)** TIGR00419 onto which HMM scores are mapped.

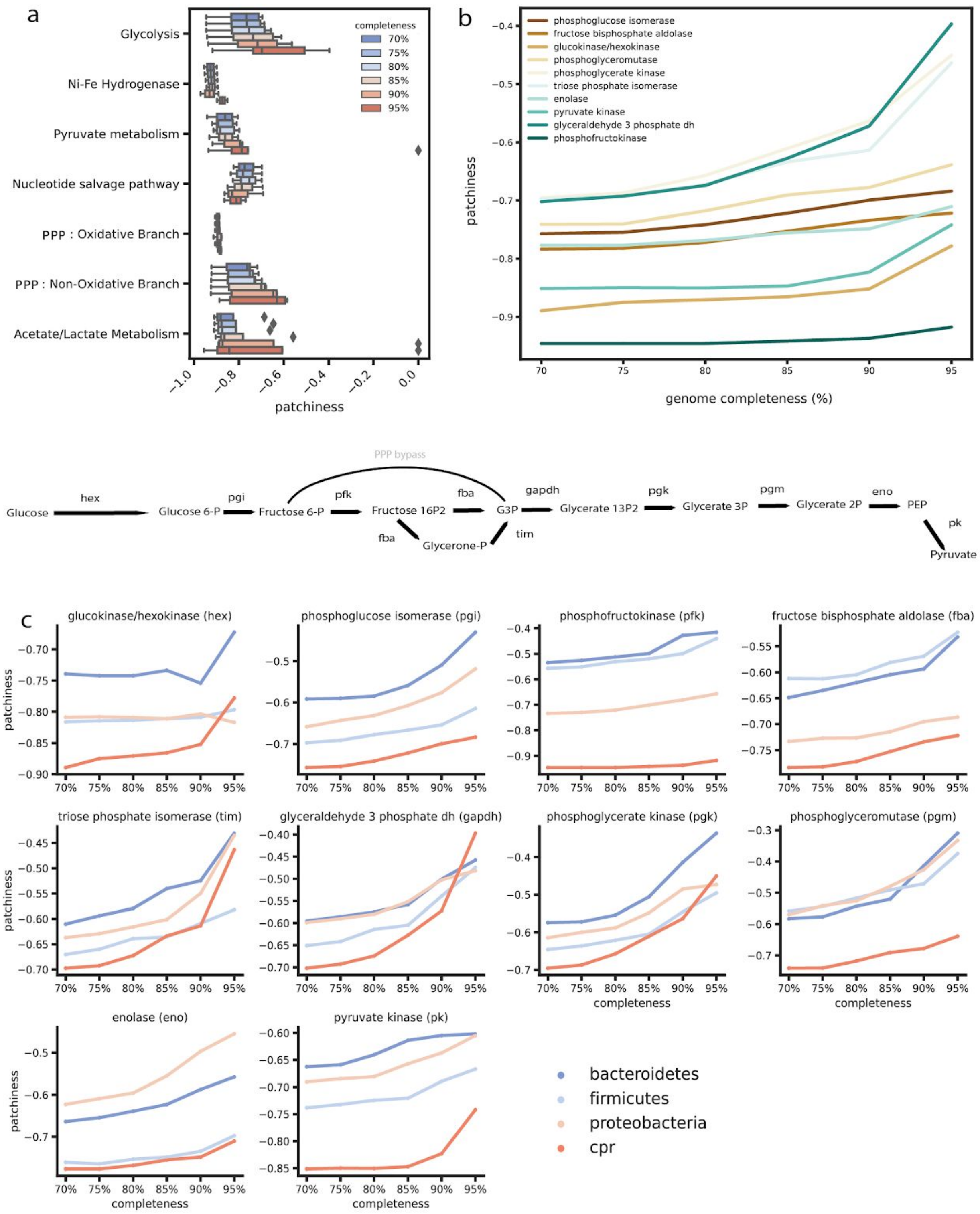

Figure S4. Impact of genome completeness on patchiness for **a)** all enzymes within the major pathways in CPR bacteria examined in this study and **b)** individual enzymes within CPR glycolysis. Part **c)** shows the patchiness of each individual glycolytic enzyme as a function of genome completeness for four major lineages, including the CPR. PPP=Pentose Phosphate Pathway. dh = dehydrogenase.

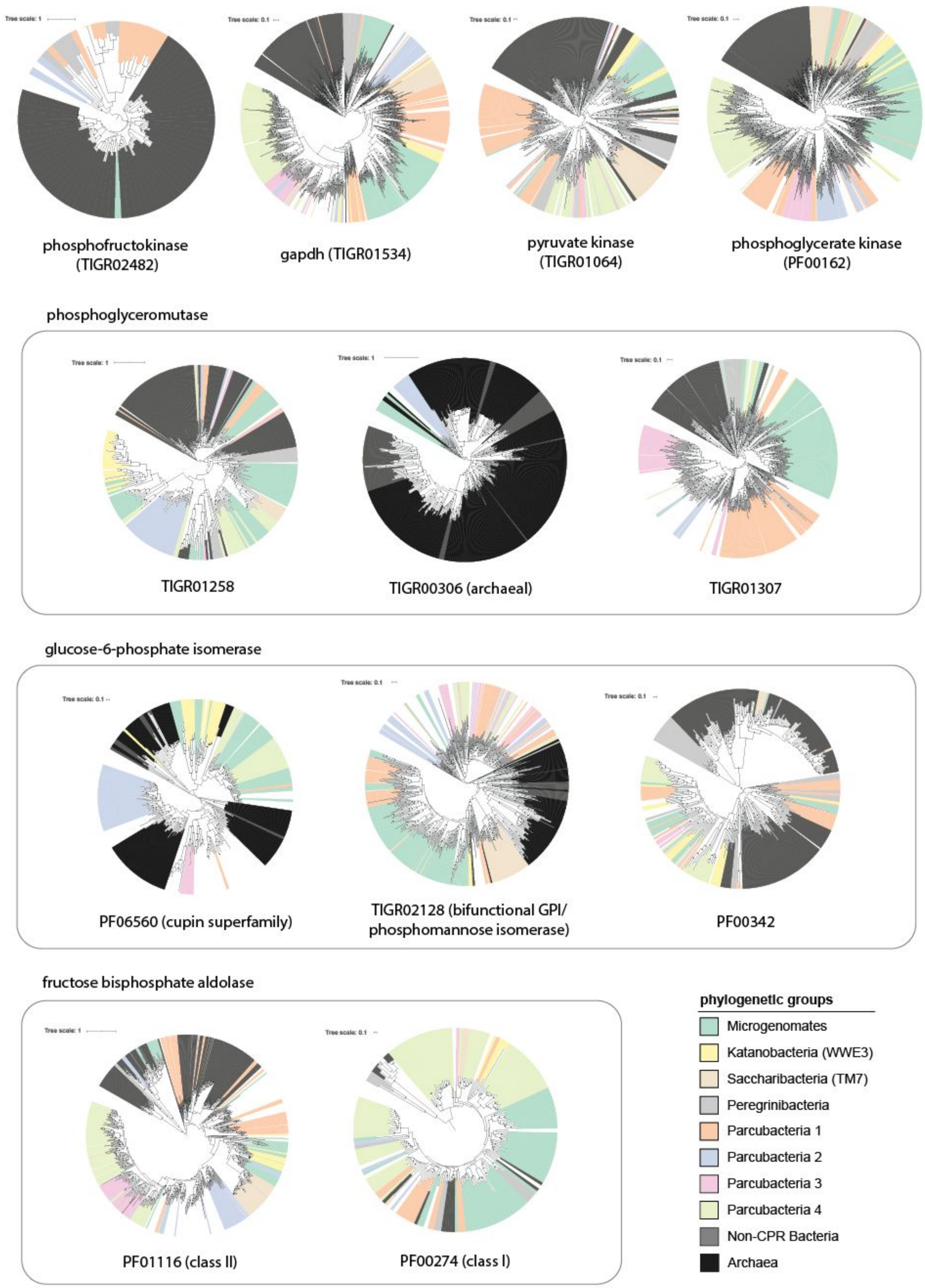

Figure S5. Maximum-likelihood gene trees for glycolytic enzymes in CPR bacteria. Different HMMs representing the same functions are grouped together by boxes. Scale bars represent the average number of substitutions per site. Black dots indicate tree nodes with >=95% ultrafast bootstrap support.

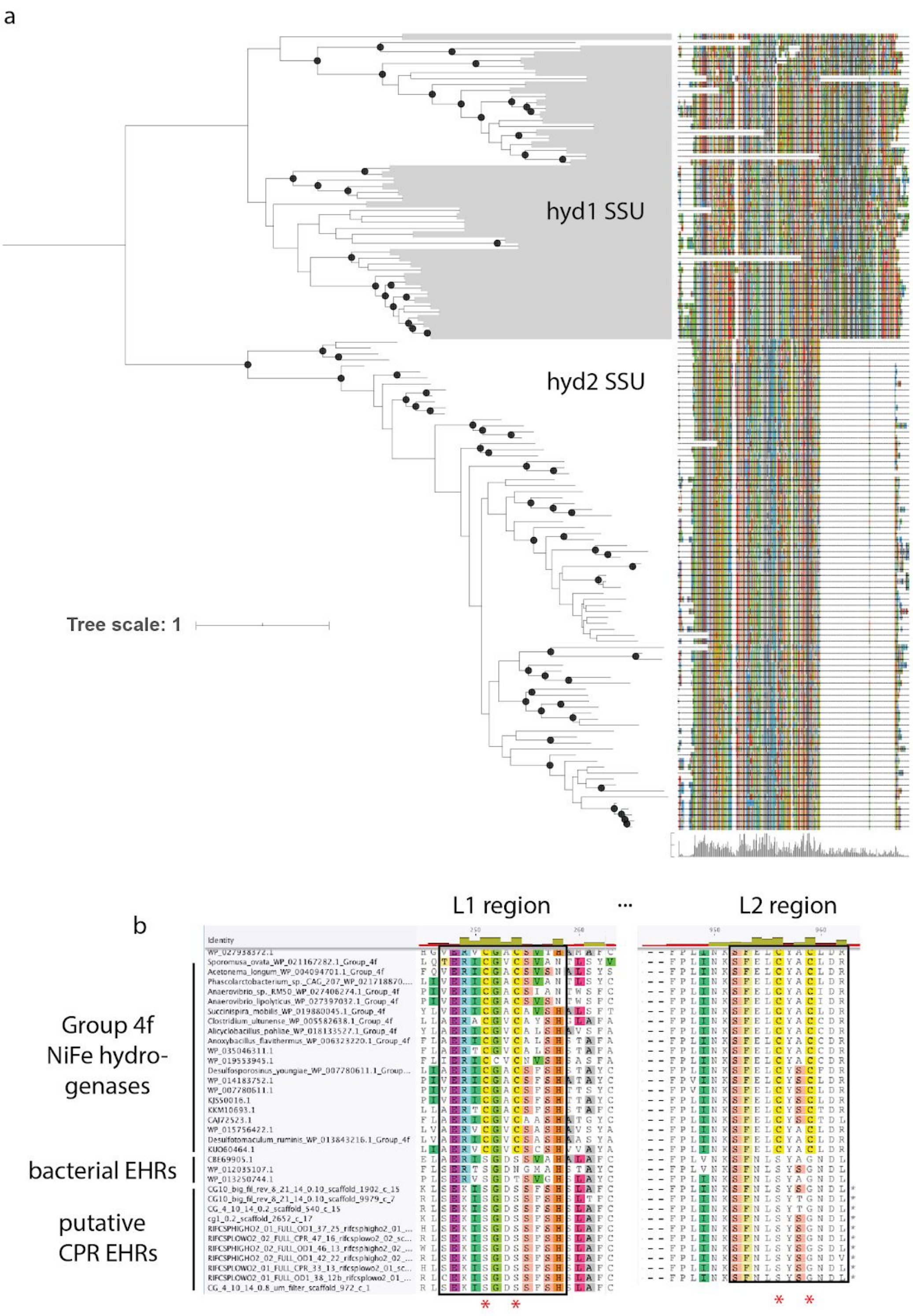

Figure S6: a) Maximum-likelihood gene tree for 3b-related NiFe hydrogenase small subunit (SSU) (fam019) with trimmed protein alignment for SSU sequences. Scale bar represents the average number of substitutions per site. Black dots indicate tree nodes with >=95% ultrafast bootstrap support. **b)** Partial alignment of the L1 and L2 regions of putative Group 4-related NiFe hydrogenases. EHR = energy-converting hydrogenases-related complexes. Red asterisk indicates cysteine residues associated with metal cofactor binding. **N.B.** for visual clarity, only a subset of sequences and sites are shown.
